# Unexpected Death of a Child with Complex Febrile Seizures—Pathophysiology Similar to Sudden Unexpected Death in Epilepsy?

**DOI:** 10.3389/fneur.2017.00021

**Published:** 2017-02-01

**Authors:** Brian J. Dlouhy, Michael A. Ciliberto, Christina L. Cifra, Patricia A. Kirby, Devin L. Shrock, Marcus Nashelsky, George B. Richerson

**Affiliations:** ^1^Department of Neurosurgery, University of Iowa Hospitals and Clinics, Iowa City, IA, USA; ^2^Pappajohn Biomedical Institute, University of Iowa Carver College of Medicine, Iowa City, IA, USA; ^3^Department of Pediatrics, University of Iowa Hospitals and Clinics, Iowa City, IA, USA; ^4^Department of Pathology, University of Iowa Hospitals and Clinics, Iowa City, IA, USA; ^5^Department of Neurology, University of Iowa Hospitals and Clinics, Iowa City, IA, USA

**Keywords:** febrile seizures, epilepsy, sudden unexplained death in childhood, sudden unexpected death in epilepsy, sudden infant death syndrome, sudden death in the young, sudden unexpected infant death

## Abstract

Febrile seizures are usually considered relatively benign. Although some cases of sudden unexplained death in childhood have a history of febrile seizures, no documented case of febrile seizure-induced death has been reported. Here, we describe a child with complex febrile seizures who died suddenly and unexpectedly after a suspected seizure while in bed at night during the beginning phases of sleep. She was resuscitated and pronounced brain dead 2 days later at our regional medical center. Autopsy revealed multiorgan effects of hypoperfusion and did not reveal an underlying (precipitating) disease, injury, or toxicological cause of death. Although a seizure was not witnessed, it was suspected as the underlying cause of death based on the medical examiner and forensic pathologist (author Marcus Nashelsky) investigation, the post-resuscitation clinical findings, and multiple aspects of the clinical history. The child had a history of complex febrile seizures that had previously caused apnea and oxygen desaturation. She had two febrile seizures earlier on the same day of the fatal event. Interestingly, her mother also experienced a febrile seizure as a child, which led to respiratory arrest requiring cardiorespiratory resuscitation. This case suggests that in a child with complex febrile seizures, a seizure can induce death in a manner that is consistent with the majority of cases of sudden unexpected death in epilepsy (SUDEP). Further work is needed to better understand how and why certain individuals, with a history of epilepsy or not, die suddenly and unexpectedly from seizures. This will only occur through better understanding of the pathophysiologic mechanisms underlying epileptic and febrile seizures and death from seizures including SUDEP.

## Introduction

Febrile seizures are usually considered relatively benign, and no reported cases of febrile seizure-induced death have been documented (1, 2). By contrast, sudden unexpected death in epilepsy (SUDEP) is a well-established phenomenon but excludes patients without a diagnosis of epilepsy (3). However, in susceptible individuals, the pathophysiologic mechanisms underlying SUDEP (4) could potentially be induced by a first unprovoked seizure, or even by a provoked seizure (5). Increasing evidence suggests that some cases of sudden unexplained death in childhood (SUDC) are associated with a history of febrile seizures (6, 7).

Here, we describe a case of a child with complex febrile seizures who died suddenly and unexpectedly after a suspected seizure while in bed at night, with her face partially turned and covered, and during the beginning phases of sleep. We propose that this death may have resulted from the same pathophysiologic mechanisms as those believed to occur in the majority of SUDEP cases as detailed in MORTEMUS (8), outlined in multiple reviews (4, 5, 9) and discussed in recent studies examining the pathophysiology surrounding SUDEP (10)—a generalized tonic–clonic seizure (GTCS) leads to ictal and postictal respiratory dysfunction and hypoxemia, which is exacerbated by being facedown in bed, during sleep, and which ultimately leads to bradycardia and asystole.

## Methods

All inpatient and outpatient records were reviewed, and patient demographics, clinical history, clinical presentation, radiographic findings, medical examiner autopsy findings, and neuropathology were recorded and analyzed. Written and informed consent was obtained.

## Case Report

A 21-month-old girl with normal healthy development and without significant past medical history initially presented twice on the same day with two witnessed complex febrile GTCSs 12 months prior to her death. One seizure led to loss of breathing and oxygen desaturation with cyanosis of the lips and cyanosis and mottling of the skin. The child was febrile to 39.4°C in our emergency department following the second seizure. She was postictal, sleepy, and ataxic, with an otherwise normal examination and was admitted for observation. Her hospital course was unremarkable, and she was discharged the following day.

One month later, she had a simple febrile seizure in the setting of croup and, 7 months later, had two more GTCSs in the setting of another upper respiratory illness. One of these seizures occurred at night while in bed with her mother, who awakened to the GTCS. The child again became cyanotic with the seizure, requiring stimulation by her mother, and ultimately began breathing again in the postictal period. In all of these episodes, she quickly returned to baseline after the seizures and the febrile illnesses resolved without complication.

She continued to develop normally and, at 33 months of age, she had witnessed another GTCS. At that time, she was seen in the emergency department, where she was postictal and sleepy with a temperature of 38.2°C. She was otherwise normal on neurological examination. A few hours later, she had witnessed another GTCS with postictal drowsiness. She recovered but did not return to her baseline level of activity that day. That night, she was placed in her parents’ bed to sleep between her mother and her sister. When last seen alive by her father, she was on her left side facing her sister. Approximately 30 min later, her father saw that she was still on her left side closely adjacent to her sister, who was also on her left side. Her face was close to a pillow and her sister’s back. The father found that she was unresponsive, pulseless, and apneic.

Her mother, a certified registered nurse anesthetist, and paramedics performed cardiorespiratory resuscitation. There was eventual return of spontaneous circulation after approximately 30 min after being found unresponsive. She was transported to our hospital. Neurological examination revealed no brainstem reflexes and no movement of the extremities. A CT scan of the brain showed diffuse swelling of the cerebral hemispheres, consistent with hypoperfusion brain injury. An electroencephalogram showed severe generalized voltage suppression without clear cerebral activity. An echocardiogram revealed adequate cardiac function with a normal heart rate and blood pressure. A chest radiograph showed airspace changes in the upper lobes of the lungs. Clinical evaluation revealed no evidence of traumatic injury. She was pronounced brain dead 2 days after admission. Her parents consented to organ donation, which was followed by autopsy performed by a medical examiner (author Marcus Nashelsky).

The cause of death was determined by the medical examiner and forensic pathologist (author Marcus Nashelsky) to be “global hypoxic/ischemic encephalopathy due to prolonged, resuscitated cardiopulmonary arrest due to complications of recurrent complex febrile seizures or epilepsy.” At autopsy, no toxicological or structural underlying cause of death was identified. Neuropathological examination of the brain revealed global cerebral edema and marked softening of the brain with central, uncal, and tonsillar herniation—expected findings days after an anoxic brain injury. No masses were identified. Microscopy revealed diffuse hypoxic–ischemic neuronal injury in all areas sampled. There was no evidence of mesial temporal sclerosis, gliosis, neuronal loss, or cortical dysplasia. No histologic findings that have been associated with epilepsy were found.

Interestingly, and as a possible forewarning, when the child’s mother was a child, she had a febrile seizure that led to respiratory arrest, thereby requiring cardiorespiratory resuscitation and a weeklong hospitalization.

## Discussion

Febrile seizures are usually considered relatively benign, occur most commonly between the ages of 6 months and 6 years, have a peak incidence at 18 months, and occur in 2–5% of children before the age of 5 years (1, 2). In the case presented here, a 2.75-year-old child with a history of complex febrile GTCSs had a suspected seizure in bed at night. She was found unresponsive, pulseless, and apneic approximately 30 min after she was last checked by her father. Although a seizure was not witnessed, it was suspected based on the circumstances, clinical findings surrounding death, and aspects of the clinical history. First, the child had a history of multiple GTCSs during febrile episodes and had two febrile GTCSs on the same day she was later found unresponsive in bed. Second, the child was found with her face turned slightly downward, near her sister’s shoulder and the pillow, a position that may have limited airflow, a common finding during seizures that lead to death. Remarkably, the scenario surrounding this death is commonly found in epilepsy patients who die from SUDEP after a GTCS. However, this child did not have a diagnosis of epilepsy and therefore, by definition, cannot be called SUDEP.

Despite semantic and classification differences, the commonalities between this case and the majority of SUDEP (Table 1) cases suggest a common pathophysiologic mechanism. The MORTEMUS study (8) revealed that SUDEP cases are often induced by a GTCS while the individual is in bed, at night, often during sleep and facedown. A GTCS can lead to activation/inactivation of brain regions that are functionally connected to the brainstem respiratory network (4), which can result in ictal and postictal respiratory dysfunction and oxygen desaturation. Recent work by Dlouhy et al. (10) found that seizure spread to the amygdala as well as electrical stimulation of the amygdala resulted in apnea and oxygen desaturation in humans. Being prone, facedown in bed, and asleep would likely exacerbate this oxygen desaturation. This hypoxemia may ultimately lead to bradycardia, asystole, and death. The fact that many SUDEP cases are found prone in bed suggests that this position plays a role in the pathophysiology. The prone position where the face may be covered by pillows or blankets likely predisposes to impaired ventilation and oxygenation during inhibition of respiratory motor output. This position also suggests a loss of arousal by patients, as they do not sense the alarm of rising CO_2_ concentrations (4). Mechanistically, this loss of arousal during the peri-ictal period may be explained by an especially striking observation in the study by Dlouhy et al. (10) in which apnea evoked by amygdala stimulation was not accompanied by dyspnea. In fact, the patients were completely unaware that they had stopped breathing. Therefore, a loss of arousal to CO_2_ may occur with amygdala seizures.

**Table 1:** **Shared and different characteristics between SUDC, SUDEP, and case presented here**.

Clinical feature	SUDC	SUDEP	Case
Occurrence at night	Yes	Yes	Yes
Occurrence during sleep	Yes	+/−	Yes
Prone positioning	Yes	Yes	Yes
Facedown	Yes	Yes	Yes
History of febrile seizures	Yes	+/−	Yes
History of GTCS	Yes	Yes	Yes
Suspected respiratory dysfunction at death	Unknown	Yes	Suspected
History of apneic seizures	Unknown	Unknown	Yes
Apparent lack of struggle at death	Yes	Yes	Yes
History of epilepsy	No	Yes	No
Age at death	1–5 years	Any age	2.75 years

*SUDC, sudden unexplained death in childhood; SUDEP, sudden unexpected death in epilepsy; GTCS, generalized tonic–clonic seizure*.

The child discussed here was known to have apnea (witnessed and described by the patient’s mother who is an experienced health-care provider) during her febrile seizures with oxygen desaturation causing cyanosis, further supporting the hypothesis that a seizure in this case may have led to a cascade of events similar to what is seen in SUDEP cases. Strikingly, and supporting the likelihood that a seizure led to death in this case, the mother of the child also experienced a febrile seizure in childhood, which led to respiratory arrest requiring cardiorespiratory resuscitation and prolonged hospitalization.

Increasing evidence, as discussed above, suggests peri-ictal respiratory dysfunction as the primary cause for SUDEP. However, death still may have occurred through other mechanisms in this child. Although evidence suggests a seizure occurred immediately prior to death, it is possible that a seizure immediately proceeding death did not occur. Prolonged autonomic effects from the two complex febrile seizures occurring earlier in the day could have resulted in cardiac changes or arrhythmia at night resulting in death.

We acknowledge that this is a rare case. However, in a large population-based cohort of children, Vestergaard et al. (11) found that mortality is increased in children during the 2 years after having a complex febrile seizure. Additionally, retrospective analysis of 121 cases of SUDC as part of the San Diego SUDC research project found that 48.8% of SUDC cases had a personal and/or family history of febrile seizures and therefore was a significant risk factor for SUDC (7). In another retrospective review of 123 consecutive children with SUDC reported to the SUDC program of the SUDC Foundation, 31.7% of SUDC cases had a personal history of febrile seizures (6). An ongoing prospective SUDC registry may help support the hypothesis that febrile seizures can lead to death with similar pathophysiology to SUDEP and how commonly this occurs.

The cause of febrile seizures is unclear and likely multifactorial. No single susceptibility gene has been found to cause febrile seizures (12). However, some genetic mutations that result in epilepsy syndromes have a component of recurrent febrile seizures (2). Mutations in SCN1A, a sodium ion channel, can result in both generalized epilepsy with febrile seizures plus (GEFS+) and Dravet syndrome (DS) (2, 12). The risk of SUDEP is high in children with DS, probably more than other infantile epilepsies (13). It is unclear why patients with DS have a high rate of early mortality but it may be related to the severity and frequency of the seizures (5). Mutations in SCN1B are linked to GEFS+, temporal lobe epilepsy, and DS (14–16). Further work in linking SCN1B and other genes encoding ion channels in the brain to SUDEP are needed. The child in this case did not fit the clinical picture of having an epilepsy syndrome with febrile seizures.

As further progress is made in defining the mechanisms involved in SUDEP and SUDC, more of these cases will be “explained” with respect to pathophysiologic causes. Mechanistic overlap likely exists, and further evidence may support that some cases of SUDC are due to unrecognized seizures, and some cases of sudden death are induced by provoked seizures. As that happens, these categorical terms will begin to lose meaning and hinder our ability to accurately describe the cause of death—therefore, as we learn more, it will be more rational to classify these deaths based on pathophysiologic mechanism.

## Conclusion

This case suggests that febrile seizures can lead to sudden unexpected death in children through mechanisms similar to those involved in SUDEP. Further study is needed to better understand how seizures of any kind, including provoked and epileptic, cause death. As we learn more, it will become possible to divide SUDC into subgroups based on pathophysiologic mechanism.

## Ethics Statement

The parents of the child described here consented to this study and report.

## Author Contributions

Author contributions to the study and manuscript preparation include the following. Conception and design: BD, MN, and GR. Acquisition of data, and analysis and interpretation of data: all the authors. Draft of the article: BD. Critical revision of the article and review of submitted version of the manuscript: all the authors. Approval of the final version of the manuscript on behalf of all the authors, administrative/technical/material support, and study supervision: BD.

## Conflict of Interest Statement

The authors report no conflict of interest concerning the materials or methods used in this study or the findings specified in this paper.
